# Comparison of the effectiveness of high-flow nasal oxygen vs. standard facemask oxygenation for pre- and apneic oxygenation during anesthesia induction: a systematic review and meta-analysis

**DOI:** 10.1186/s12871-022-01615-7

**Published:** 2022-04-06

**Authors:** Jian-li Song, Yan Sun, Yu-bo Shi, Xiao-ying Liu, Zhen-bo Su

**Affiliations:** Department of Anesthesiology, China-Japan Union Hospital of Jilin University, No. 126, Xiantai Rd, Changchun, 130000 China

**Keywords:** High flow nasal oxygen, Facemask ventilation, Preoxygenation, Anesthesia induction, Airway management

## Abstract

**Background:**

In recent years, high flow nasal oxygen (HFNO) has been widely used in clinic, especially in perioperative period. Many studies have discussed the role of HFNO in pre- and apneic oxygenation, but their results are controversial. Our study aimed to examine the effectiveness of HFNO in pre- and apneic oxygenation by a meta-analysis of RCTs.

**Methods:**

EMBASE, PUBMED, and COCHRANE LIBRARY databases were searched from inception to July 2021 for relevant randomized controlled trails (RCTs) on the effectiveness of HFNO versus standard facemask ventilation (FMV) in pre- and apenic oxygenation. Studies involving one of the following six indicators: (1) Arterial oxygen partial pressure (PaO_2_), (2) End expiratory oxygen concentration (EtO_2_), (3) Safe apnoea time, (4) Minimum pulse oxygen saturation (SpO_2min_), (5) Oxygenation (O_2_) desaturation, (6) End expiratory carbon dioxide (EtCO_2_) or Arterial carbon dioxide partial pressure(PaCO_2_) were included. Due to the source of clinical heterogeneity in the observed indicators in this study, we adopt random-effects model for analysis, and express it as the mean difference (MD) or risk ratio (RR) with a confidence interval of 95% (95%CI). We conducted a risk assessment of bias for eligible studies and assessed the overall quality of evidence for each outcome.

**Results:**

Fourteen RCTs and 1012 participants were finally included. We found the PaO_2_ was higher in HFNO group than FMV group with a MD (95% CI) of 57.38 mmHg (25.65 to 89.10; *p* = 0.0004) after preoxygenation and the safe apnoea time was significantly longer with a MD (95% CI) of 86.93 s (44.35 to 129.51; *p* < 0.0001) during anesthesia induction. There were no significant statistical difference in the minimum SpO_2_, CO_2_ accumulation, EtO_2_ and O_2_ desaturation rate during anesthesia induction between the two groups.

**Conclusions:**

This systematic review and meta-analysis suggests that HFNO should be considered as an oxygenation tool for patients during anesthesia induction. Compared with FMV, continuous use of HFNO during anesthesia induction can significantly improve oxygenation and prolong safe apnoea time in surgical patients.

**Supplementary Information:**

The online version contains supplementary material available at 10.1186/s12871-022-01615-7.

## Introduction

Oxygenaton is fundamental to safe anaesthetic practice and anaesthesiologists need to be skilled in oxygenation techniques. Hypoxemia during anesthesia induction is one of the leading causes of anesthesia-related morbidity and mortality [1], and anesthesiologists should take it seriously and avoid its occurrence. It has been reported that cardiac arrests can occur in 2–3% of intubation procedure in intensive care unit (ICU), and is strongly related to hypoxemia or absence of preoxygenaion before intubation [2]. Preoxygenation before anesthesia induction can increase alveolar oxygen reserve of patients by denitrogenation, so as to increase safe apnoea time and reduce the incidence of hypoxemia and subsequent complications during endotracheal intubation. Consequently, the Difficult Airway Society guidelines recommended that all patients should be preoxygenated before induction of general anesthesia [3]. The standard method of preoxygenation is performed using a facemask with an adequate seal between the patient and the circuit for 3 min with a fresh gas flow of 10 L·min^−1^[4]^.^In addition, apneic oxygenation can also prolong safe apnoea time and reduces the incidence of arterial oxygen desaturation during intubation [5]. Preoxygenation and apneic oxygenation are especially important in patients whereby bag-mask ventilation after the induction of anesthesia is to be avoided and in patients at higher risk of hypoxemia [5, 6].

HFNO is composed of an air/oxygen blender, an active humidifier, a single heated circuit and a nasal cannula, which can provide constant inhaled oxygen concentration of 0.21–1.0 and oxygen flow rate of 1–60 L·min^−1^ or even higher [7]. It has been proposed that the use of HFNO can generate continuous positive airway pressure, reduce anatomical dead space, improve mucociliary clearance and reduce the work of breathing [8–11]. In 2015, HFNO was first used for preoxygenation and apneic oxygen in patients with predicted difficult airway, and was proposed that HFNO can significantly prolong the safe apnoea time of patients under general anesthesia [6]. Many clinical anesthesiologists has carried out extensive and in-depth research on the application of HFNO in perioperative period, especially in the pre- and apneic oxygenation efficacy of HFNO during anesthesia induction. However, many studies have reached controversial results. There was a systematic review and meta-analysis have indicated the use of HFNO in the intraoperative setting can reduce the risk of O_2_ desaturation, increase safe apnoea time and SpO_2min_ in patients at higher risk of hypoxemia [12]. However, it was based on small-sampled studies and did not restrict the control group to standard face mask ventilation. In addition, recent published RCTs can be included in our systematic and meta-analysis [13–19].

Therefore, we conducted a systematic review and meta-analysis to update the existing evidence and gain further insight into the effectiveness of HFNO compared with FMV for pre- and apneic oxygenation during anesthesia induction. We selected 6 indicators to compare the use of HFNO and FMV during anesthesia induction. Among them, PaO_2_ and EtO_2_ can represent the efficacy of pre-oxygenation, safe apnoea time, SpO_2min_ and O_2_ desaturation can represent the efficiency of pre-oxygenation, and EtCO_2_ or PaCO_2_ can be used to observe the effect of apneic oxygenation on patient ventilation [4].

## Methods

### Search strategy

This systematic review and meta-analysis was conducted in accordance with the Preferred Reporting Items for Systematic Review and Meta-Analysis (PRISMA) guidelines [20]. The PRISMA Checklist is provided in Additional file 1. English databases including PUBMED, EMBASE, and COCHRANE LIBRARY were searched from inception to December 2021 to find RCTs exploring the effectiveness of HFNO compared with FMV for pre- and apneic oxygenation in adult patients (> 18 years old). According to the PICOS approach, the following terms were selected: “High flow nasal oxygen,””HFNO,” “High flow nasal cannula,” “HFNC,” “Transnasal humidified rapid-insufflation ventilatory exchange,” “THRIVE,” “Facemask,” “Facemask ventilation,” “Preoxygenation,” “Intubation,” “Anesthesia induction,””Randomised controlled trial,” “RCT,” “randomized,” “controlled,”. We also searched Google Scholar and clinical trail registry to identify grey literature and checked the reference list of all included studies to identify additional studies missed from the original electronic search. This study does not contain any conference abstracts.

### Inclusion and exclusion criteria

Inclusion criteria were as follows: 1)comparing the effects of HFNO and FMV during anesthesia induction; 2)involving one of the following six indicators: (1) PaO_2_, (2) EtO_2_, (3) safe apnoea time, (4) SpO_2min_, (5) O_2_ desaturation, (6) EtCO_2_ or PaCO_2_, at anesthesia induction period for pre- or apenic oxygenation; 3) randomized controlled trials. We excluded studies if they 1) were intensive care unit and pediatric patients; 2)were non mask controlled experiments, including bite block or nasal cannula ventilation; 3)were not able to extract data; 4) were not available for full text.

### Articles selection and data extraction

Titles and abstracts were independently screened by 2 authors (Song, Sun). Following selection of abstracts, full text of articles identified for possible inclusion were obtained and assessed for inclusion independently by the 2 reviewers (Song, Sun). Disagreements were resolved by consensus or by consulting the senior author (Su). Study characteristics were extracted independently by 2 authors (Shi, Liu) using a standard data collection form in an Excel worksheet. The following information was extracted from each study: author, year of publication, type of surgery, number of patients, intervention characteristics and inclusion indicators. The 6 indicators extracted were PaO_2_, EtO_2_, safe apnea time, SpO_2min_, O_2_ desaturation and EtCO_2_ or PaCO_2_. The data were extracted independently by two authors (Shi, Liu) and then reviewed by the senior author (Su). When there is missing data, contact the relevant author to obtain the missing data.

When comparing the safe apnoea time, the included articles have different definitions. Two defined from the cessation of spontaneous breathing until the SpO_2_decreased to 90% or the apnoea time reached 6 min or 10 min [13, 14], one defined the apnoea time from the onset of cessation of breathing until the SpO_2_ decreased to 95% or the apnoea time reached 6 min [21] and one defined from the cessation of spontaneous breathing until the SpO_2_decreased to 92% [18]. And there are also differences in the definition of deoxysaturation, desaturation was defined as SpO_2_≦90% in two studies [22, 23], SpO_2_≦93% in two studies [15, 24] and SpO_2_≦92% in one study [18].  In our analysis, we directly compared this indicator without adopting a unified definition.

### Risk of bias assessment

Two reviewers (Song, Sun) independently assessed risk of bias in included studies using the Cochrane Collaboration risk-of-bias tool [25]. Studies were categorized into high, low, or unclear risk of bias according to the following predefined criteria: random sequence generation (selection bias), allocation concealment (selection bias), blinding of participants and personnel (performance bias), blinding of outcome assessment (detection bias), incomplete outcome data (attrition bias), selective reporting (reporting bias), and other potential sources of bias. Each study was compared for consistency, with any disagreement resolved by discussion between the two reviewers (Song, Sun) or mediated by a third reviewer (Su).

### Statistical analysis

Meta-analysis was performed using Review Manager (RevMan version 5.4.1, The Nordic Cochrane Centre, Copenhagen, Denmark). Categorical and continuous variable summary data from each individual study were entered into Review Manager. The statistical method used for categorical outcome (O_2_ desaturation) was Mantel–Haenszel and the effect measure was risk ratio (RR). The statistical method used for continuous outcome (PaO_2_, EtO_2_, safe apnoea time, SpO_2min_, EtCO_2_ or PaCO_2_) was inverse variance and the effect measure was mean difference. Due to the source of clinical heterogeneity in the observed indicators in this study, we adopt random-effects model for analysis. Subgroup analysis and sensitivity analysis excluding literature one by one were used to explore the causes of high heterogeneity. Forest plots, RR (95% confidence interval [CI]), mean difference (95% CI), and heterogeneity (*χ*^*2*^ and *I*^*2*^) were generated for the 6 outcomes. For studies that showed results in median and range or interquartile range, the methodology of Wan et al. [26] was used to convert them into mean and standard deviation.

## Results

**Fig. 1:**
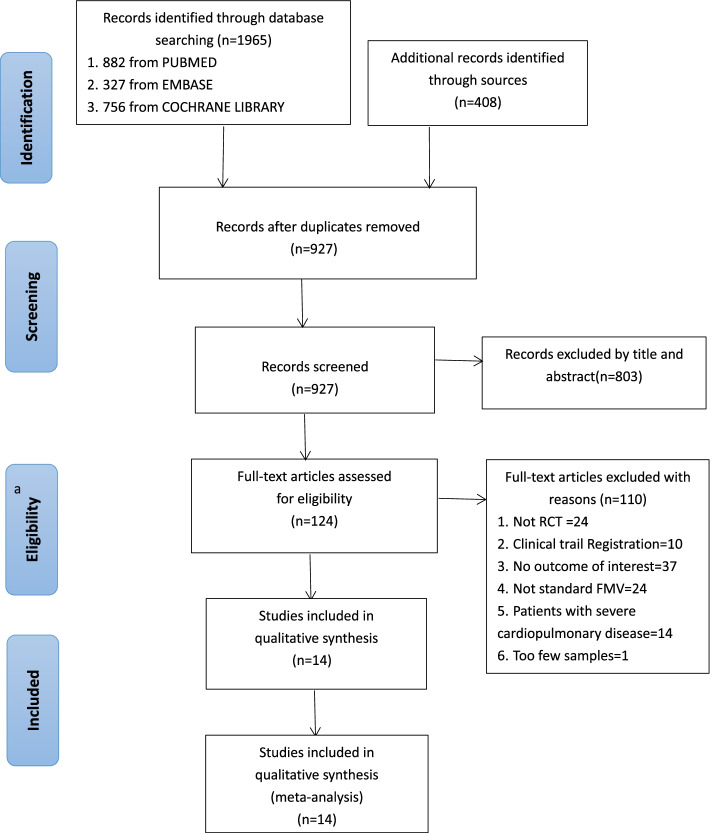
Study flow diagram of trial selection

**Fig. 2:**
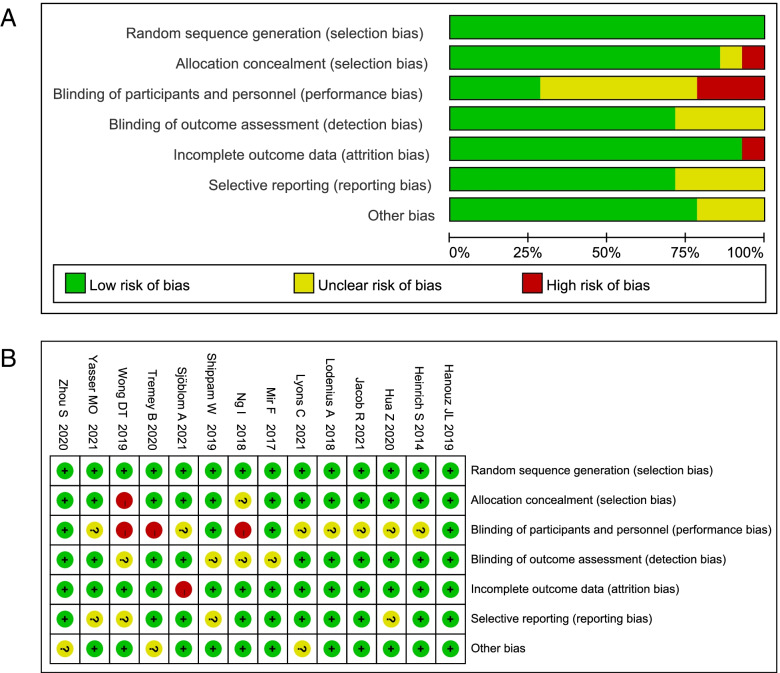
Risk of bias assessment. **A** risk of bias summary. **B **Risk of bias graph. The plus sign indicates low risk, the sinus sign indicates high risk, and the question sign mark uncertain risk

The initial electronic search retrieved 1965 citations, and the grey literature search identified additional 408 studies. This process identified 121 potentially eligible studies for full-text review. After duplicate and ineligible studies were removed, 14 RCTs with a total of 1012 participants were finally included in our systematic review and meta-analysis (Fig. 1) [13–19, 21–24, 27–29]. The characteristics of included studies are presented in Table 1. The methodological quality of the involved trails is shown in Fig. 2. Two studies were multi-center RCT [15, 17] and the reminder were single-center RCTs. All 14 studies included one or more of the following outcomes: (1) PaO_2_, (2) EtO_2_, (3) safe apnoea time, (4) SpO_2min_, (5) O_2_ desaturation, (6) EtCO_2_ or PaCO_2_, at anesthesia induction period for pre- or apenic oxygenation.

**Table 1:**
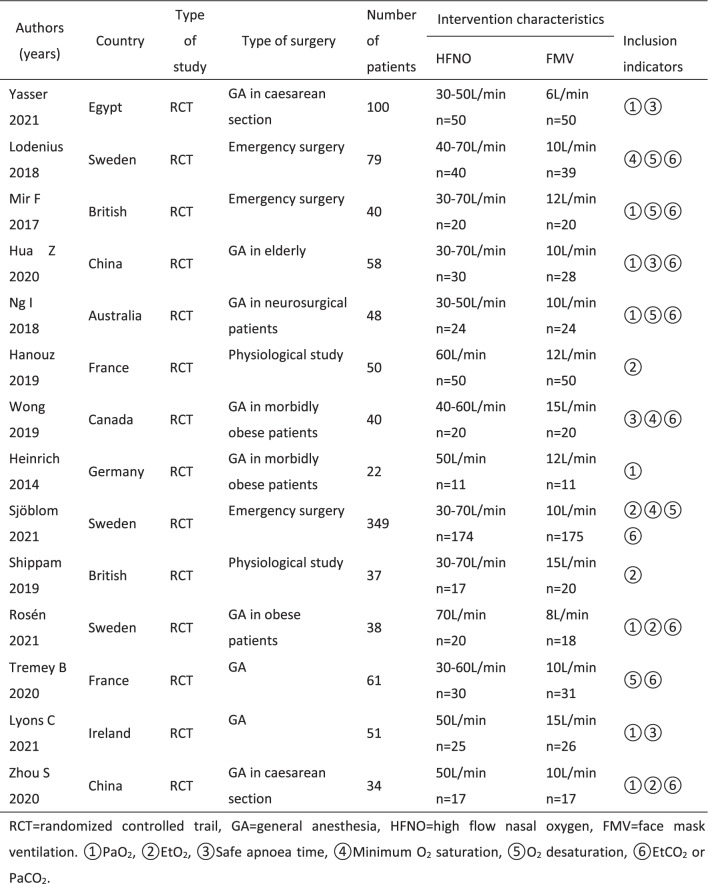
Main characteristics of included studies.

### PaO_2_

**Fig. 3:**
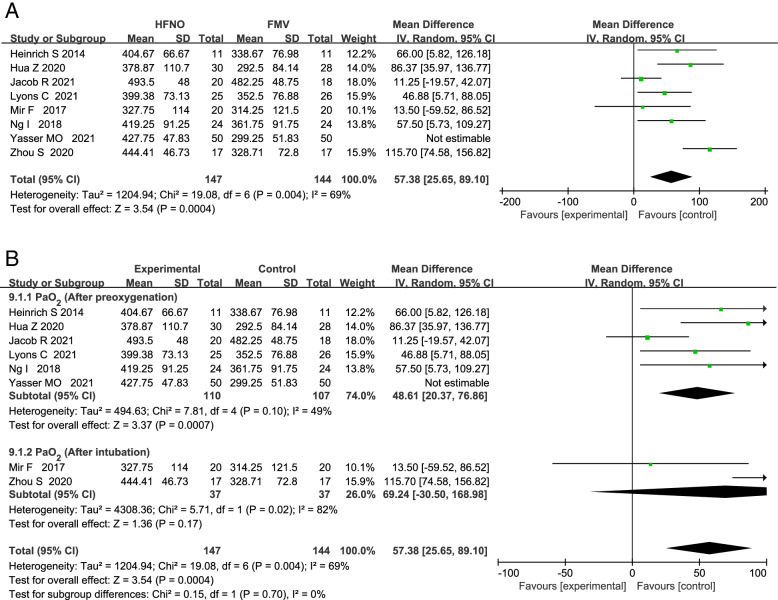
Forest plots of PaO2 in HFNO versus FMV after preoxygenation and after intubation. Subgroup analysis shows the PaO2 after preoxygenation versus after intubation. CI indicates confidence interval; df, degrees of freedom; HFNO, a high-flow nasal oxygen; FMV, facemask ventilation; IV, inverse variance, 02 oxygen, standard deviation

Eight RCTs compared the PaO_2_ after preoxygenation between HFNO and FMV group, including a total of 391 patients. HFNO was administered at flow rates between 30 and 70 L·min^−1^ while the flow rate of FMV group was 6–15 L·min^−1^ during preoxygenation. Meta-analysis based on the eight studies showed a statistically significant higher PaO_2_ after preoxygenation in the HFNO group than FMV group with a MD (95% CI) of 67.82 mmHg (29.25 to 106.40; *p* = 0.0006). Due to high heterogeneity, we performed the sensitivity analysis by excluding the eight studies one by one, and found that by excluding Yasser MO et al.’s article could significantly reduce heterogeneity. And still statistically significant with a MD (95% CI) of 57.38 mmHg (25.65 to 89.10; *p* = 0.0004; Fig. 3). The seven RCTs included a total of 291 patients. The source of heterogeneity may come from different patient populations, different pre-oxygenation time and different ways to use HFNO in the included articles. Subgroup analysis showed no significant difference in PaO2 between after preoxygenation and after intubation (*p* = 0.70; Fig. 3). Funnel plot analysis suggested visually no significant asymmetry, suggesting a low chance of publication bias (Additional file 2 and S1).

### EtO_2_

**Fig. 4:**
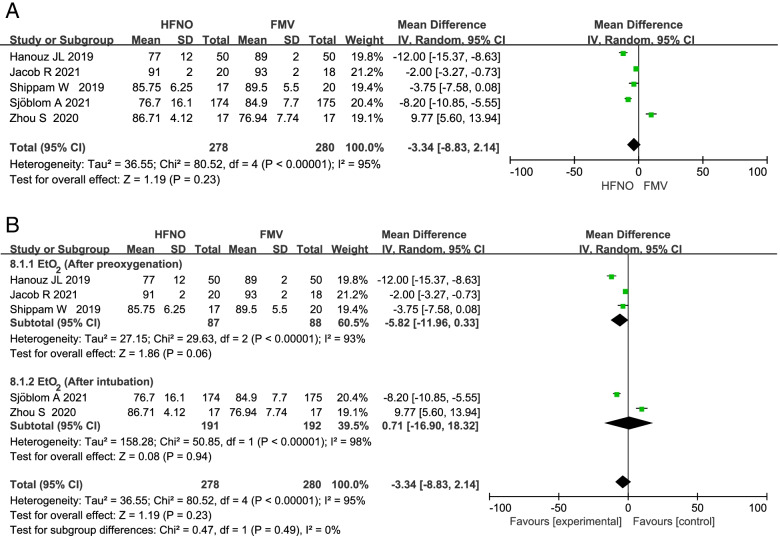
Forest plots of ETO2 in HFNO versus FMV after preoxygenation and intubation. Subgroup analysis shows the ETO2 after preoxygenation versus after intubation. CI indicates confidence interval; df, degrees of freedom; HFNO, hign flow anasal oxygen; FMV, facemask ventilation; IV, inverse variance; o2 oxygen; SD, standard deviation

Five studies compared the EtO_2_ between HFNO and FMV group. Meta-analysis based on the five studies showed that EtO_2_ was similar in the HFNO group versus FMV group with a MD (95% CI) of -3.34% (-8.83 to 2.14; *p* = 0.23; Fig. 4). Due to high heterogeneity, we performed the sensitivity analysis by excluding the five studies one by one, but there was no significant change in heterogeneity. The source of heterogeneity may come from different patient populations, different pre-oxygenation time and different ways to use HFNO in the included articles. Three studies [16, 27, 29] compared the EtO_2_ after preoxygenation and two studies [15, 19] compared the EtO_2_ after intubation. Subgroup analysis showed that there was no significant difference in EtO2 between after preoxygenation and intubation (MD -5.82; 95%CI -11.96 to 0.33; *p* = 0.06 and MD 0.71; 95%CI -16.90 to 18.32; *p* = 0.94; Fig. 4).

### Safe apnea time

**Fig. 5:**
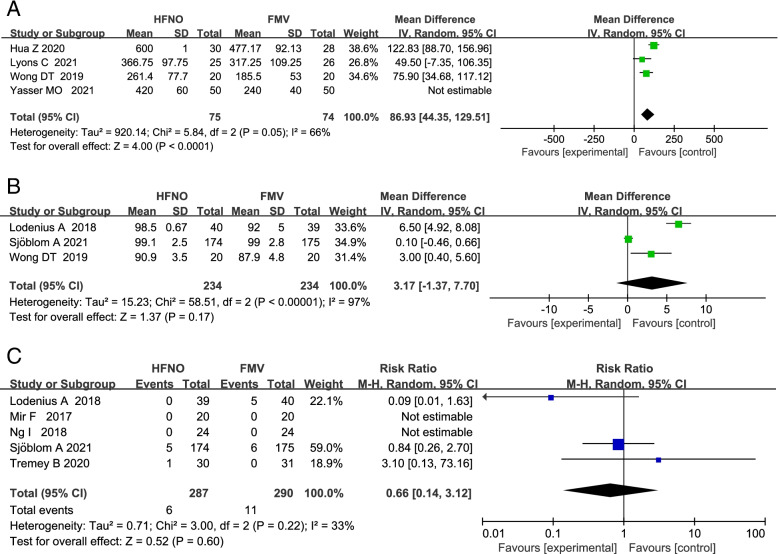
**A** Forest plots of safe apnea time in HFNO versus FMV after preoxygenation and intubation. **B** Forest plots of SpO_2_min in HFNO versus FMV during intubation. **C** Forest plots of the rate of desaturation in HFNO versus FMV during intubation. CI indicates confidence interval; MD, mean difference; RR, risk ratio; df, degree of freedom; HFNO, high flow nasal oxygenation; FMV, face mask ventilation; IV, inverse variance; O2, Oxygenation; SD, standard deviation

Four RCTs compared safe apnoea time during the peri-intubation period between HFNO and FMV. In all four RCTs, facemask assisted ventilation was not implemented in control groups during apneic oxygenation. Airway patency was carefully maintained using a chin left or jaw thrust in all subjects. From meta-analysis of the four RCTs, safe apnoea time was significantly longer in HFNO compared with FMV group by a MD (95% CI) of 110.36 s (50.56 to 170.16; *p* = 0.0003). Due to the high heterogeneity, we excluded the literature one by one for sensitivity analysis. We found that when excluding Yasser MO et al.'s research can significantly reduce heterogeneity, and there were still statistical differences with a MD (95% CI) of 86.93 s (44.35 to 129.51; *p* < 0.0001; Fig. 5A). The source of heterogeneity may come from different patient populations, different pre-oxygenation time, different ways to use HFNO and the different definitions of the safe apnoea time in the included articles.

### Minimum O2 saturation(SpO2min)

Three RCTs compared the SpO_2min_ during the peri-intubation period between HFNO and FMV. Meta-analysis showed that the SpO_2min_ was similar in HFNO and FMV subjects with a MD (95% CI) of 3.17% (-1.37 to 7.70; *p* = 0.17; Fig. 5B). Due to the high heterogeneity, we excluded the studies one by one for sensitivity analysis. After excluding Sjöblom A et al.’s study, the heterogeneity decreased slightly, but there was a significant statistical difference in HFNO verses FMV with a MD (95% CI) of 4.91% (1.49 to 8.32; *p* = 0.005). The source of heterogeneity may come from different patient populations, different pre-oxygenation time and different ways to use HFNO in the included articles.

### O_2_ desaturation

Five RCTs compared the rate of O_2_ desaturation during intubation period between HFNO and FMV group. Meta-analysis showed that the rate of peri-intubation O_2_ desaturation was similar in HFNO group versus FMV group with a RR (95% CI) of 0.66 (0.14 to 3.12; *p* = 0.60; Fig. 5C). The source of heterogeneity may come from different patient populations, different defination of desaturation, different pre-oxygenation time and different ways to use HFNO in the included articles.

### PaCO_2_ or end-tidal CO_2_

**Fig. 6:**
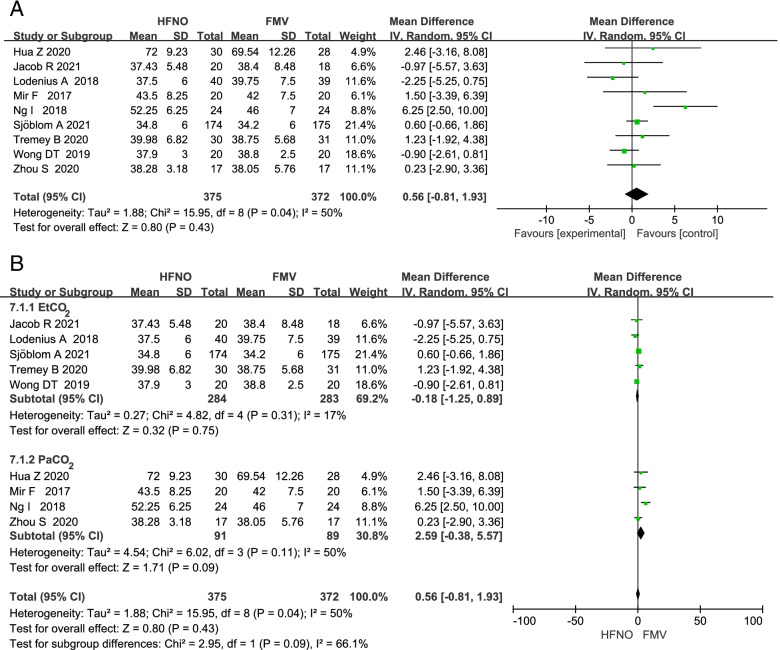
Forest plots of EtCO2 or PaCO2 in HFNO versus FMV after intubation. Subgroup analysis shows the EtCO2 versus PaCO2 after intubation. CI indicates confidence interval; df, degrees of freedom; HFNO, high-flow nasal oxygen; FMV, facemask ventilation; IV, inverse variance; O2, oxygen; SD, standard deviation

Nine RCTs compared the EtCO_2_ or PaCO_2_ between HFNO group and FMV group during intubation period. Since both EtCO_2_ and PaCO_2_ can reflect the accumulation of CO_2_ in the body, we analyzed EtCO_2_ and PaCO_2_ together. Meta-analysis showed that the CO_2_ accumulation was similar in HFNO group versus FMV group with a MD (95% CI) of 0.56 mmHg (-0.81 to 1.93; *p* = 0.43; Fig. 6). We also performed subgroup analysis with EtCO_2_ and PaCO_2_, and found no significant statistical difference (*p* = 0.09) between the EtCO_2_ group (MD -0.18; 95% CI -1.25 to 0.89; *p* = 0.75) and the PaCO_2_ group (MD 2.59; 95% CI -0.38 to 5.57; *p* = 0.09; Fig. 6). The source of heterogeneity may come from the difference between EtCO_2_ and PaCO_2_, the different pre-oxygenation time and the different apneic oxygenation time in the included articles. Funnel plot analysis suggested visually no significant asymmetry, suggesting a low chance of publication bias (S2).

## Discussion

This systematic review and meta-analysis shows that compared with FMV, HFNO can significantly improve oxygenation and prolong safe apnoea time during anesthesia induction, but there is no significant statistical difference in the rate of O_2_ desaturation, EtO_2_, minimum SpO_2_ and CO_2_ level.

Meta-analysis showed that compared with FMV group, PaO_2_ in HFNO group was higher during anesthesia induction (*p* = 0.0004) and subgroup analysis showed that there was no significant difference (*p* = 0.70) in PaO2 between after preoxygenation and after intubation. This finding shows that compared with FMV, the use of HFNO during anesthesia induction can significantly improve the oxygenation of patients, which has been confirmed by previous studies. The oxygenation efficacy of HFNO in awake fibre-optic intubation in patients with difficult airways has been studied and found that HFNO can significantly improve oxygenation and prolong the safe apnoea time [30]. Previous studies have shown that HFNO can provide a stable inspired oxygen concentration, the distal positive airway pressure generated by high flow gas can increase end-expiratory lung volume, alveolar oxygen partial pressure and reduce intrapulmonary shunt, and the less dead space ventilation than FMV due to the washout effect of THRIVE [9, 19, 23, 28]. This may be a potential mechanism for HFNO to increase the PaO_2_ compared with FMV during pre-oxygenation.

In addition, we found that safe apnoea time during anesthesia induction was longer in HFNO group than FMV group (*p* < 0.0001). This finding is in line with the previous research conclusions in both ICU and operating room [5, 31, 32]. It has been reported that HFNO can significantly prolong safe apnoea time when used for preoxygenation and apneic oxygenation during surgery in patients with predictable difficult airway, with a median apnoea time of 14 min and a maximum of 65 min [6].  HFNO can provide continuous supply for patients with apnoea through the effect of apneic oxygenation during intubation period, so as long to prolong safe apnoea time [6, 9]. Taking advantage of the fact that HFNO can significantly prolong the safe apnoea time, many medical institutions have successfully carried out tubeless anesthesia, especially in short operations with shared airway such as subglottic stenosis and upper airway surgeries [33, 34]. However, studies recently published indicates that although the apneic oxygenation of HFNO can ensure the oxygenation of patients and maintain long-term tubeless anesthesia, it is easy to result in CO_2_ accumulation and respiratory acidosis when the apnoea time is greater than 30 min [35, 36]. This extends previous knowledge and has implications for the safe application of HFNO during prolonged procedures.

However, there are still some differences compared with previous studies. In our findings, there was no significant difference in the rate of O_2_ desaturation (*p* = 0.60) and the SpO_2min_ (*p* = 0.17) between HFNO and FMV subjects during intubation period. These findings are not exactly consistent with the studies on HFNO in the ICU. Previous studies have shown that the use of HFNO during endotracheal intubation can reduce the incidence of hypoxemia and increase the minimum O_2_ saturation in ICU patients [9, 35, 37]. But, an observational study showed that the use of HFNO during emergency intubation can reduce the incidence of desaturation in patients with high risk hypoxemia [38]. And there were also studies showing no differences [39–41]. The reason for this difference may be that, unlike ICU patients, surgical patients have well compensated cardiopulmonary function.

According to our findings, we can recognize that HFNO is an effective oxygenation tool in general anesthesia surgery. Oxygenation is of paramount importance in anesthesia induction period, especially in patients with difficult airways and high-risk hypoxemia patients. Previous studies have demonstrated the effectiveness of HFNO use in these specific populations [6, 9, 37]. However, in this paper, we did not compare the use of HFNO and FMV in these special populations, and therefore cannot suggest that HFNO is superior to FMV when used in these populations.

The strengths of this review include a comprehensive search strategy using major biomedical databases for published data and grey literature, and a focus on clinically relevant outcomes. Secondly, we followed a rigorous methodology. The review of eligibility criteria, data extraction, and outcome methodology assessment were all performed in duplicate with a high degree of inter-rater agreement. Thirdly, this review contained the largest number of RCTs published on this topic, which allowed outcomes to meet the optimal information size and allowed us to make more reliable inferences.

Several potential limitations are also present in this meta-analysis. First, we included 14 RCTs and observed six indicators, and there were relatively few articles included in each index, even though this is the largest number of RCTs that can be searched. Second, in this article, we included different populations into the meta-analysis. Due to the limited number of articles included in each observation index, we did not conduct subgroup analysis for different populations. Third, in this meta-analysis, although we reduced the heterogeneity through sensitivity analysis, each observation index still has heterogeneity. Finally, due to the limited articles included in each indicator, we only evaluated the publication bias of PaO_2_ and CO_2_ indicators.

## Conclusion

This systematic review and meta-analysis comprehensively evaluated the effectiveness of HFNO verses FMV for pre- and apneic oxygenation during anesthesia induction. According to our findings, compared with FMV, HFNO can improve oxygenation of patients during pre-oxygenation, and its continuous application during induction of anesthesia can significantly prolong the safe apnoea time. We suggest that HFNO should be considered as an oxygenation tool during anesthesia induction in patients undergoing general anesthesia surgery. Further well-powered RCTs should focus on comparing the effectiveness of HFNO verses FMV in special surgical populations, such as patients with hypoxemia, patients with difficult airway and pediatric patients. 

## Supplementary Information

**Additional file 1.** PRISMA checklist.

**Additional** **file 2.** **S1:** Funnel plot of PaO_2_. S2: Funnel plot of CO2 accumulation.

## Data Availability

All data generated or analysed during this study are included in this published article [and its supplementary information files]. All data are from EMBASE, PubMed and Cochrane library databases (https://www.elsevier.com/solutions/embase-biomedical-research; https://pubmed.ncbi.nlm.nih.gov/advanced/; https://www.cochranelibrary.com/).

## Abbreviations

HFNO: High flow nasal oxygen
RCTs: Randomized controlled trails
FMV: Facemask ventilation
PaO_2_: Arterial oxygen partial pressure
EtO_2_: End expiratory oxygen concentration
SpO_2__min_: Minimum pulse oxygen saturation
EtCO_2_: End expiratory carbon dioxide
PaCO_2_: Arterial carbon dioxide partial pressure
MD: Mean difference;
RR: Risk ratio
95%CI: Confidence interval of 95%
ICU: Intensive care unit
