# Supplementary figures and images for: Comparison of the effectiveness of high-flow nasal oxygen vs. standard facemask oxygenation for pre- and apneic oxygenation during anesthesia induction: a systematic review and meta-analysis
Source: BMC Anesthesiol. 2022 Apr 6;22:100. doi: 10.1186/s12871-022-01615-7 (PMC8985355; doi:10.1186/s12871-022-01615-7)

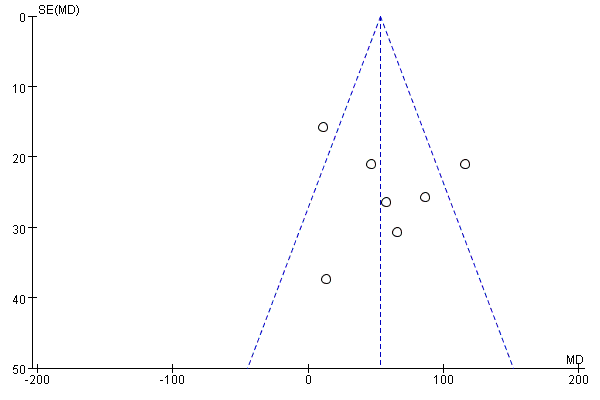


**S1: Funnel plot of PaO2**


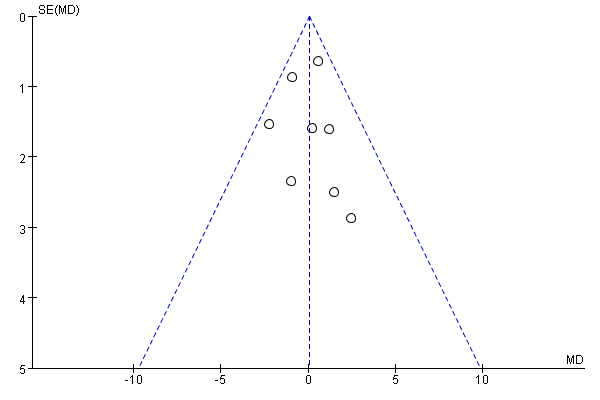


**S2: Funnel plot of CO2 accumulation.**

Supplement: Supplementary file 2 — Additional file 2. S1: Funnel plot of PaO2. S2: Funnel plot of CO2 accumulation. [file 12871_2022_1615_MOESM2_ESM.docx]
